# Burnout protective patterns among oncology nurses: a cross-sectional study using machine learning analysis

**DOI:** 10.1186/s12912-025-03277-5

**Published:** 2025-07-01

**Authors:** Ana Rocha, Cristina Costeira, Raul Barbosa, Florbela Gonçalves, Miguel Castelo-Branco, Joaquim Viana, Margarida Gaudêncio, Filipa Ventura

**Affiliations:** 1 Health Sciences Research Unit: Nursing (UICISA: E), Nursing School of Coimbra (ESEnfC), Coimbra, 3004-011 Portugal; 2 School of Health Sciences, Polytechnic University of Leiria, Campus 2, Morro do Lena, Alto do Vieiro, Apartado 4137, Leiria, 2411-901 Portugal; 3 ciTechCare - Center for Innovative Care and Health Technology, Hub de Inovação de Saúde Politécnico de Leiria Campus 5, R. das Olhalvas, Leiria, 2414-016 Portugal; 4 Centre for Informatics and Systems of the University of Coimbra (CISUC), Department of Informatics Engineering, University of Coimbra, Coimbra, Portugal; 5 Faculdade de Ciências da Saúde, Universidade da Beira Interior, Covilhã, Portugal; 6 Palliative Care Unit, Portuguese Oncology Institute of Coimbra, Coimbra, 3004 Portugal; 7 ULS Coimbra - Centro Hospitalar e Universitário de Coimbra EPE, Coimbra, Portugal

**Keywords:** Burnout, Oncology nursing, Protective factors, Work environment, Machine learning, Occupational health

## Abstract

**Background:**

Oncology nurses face unique and intense demands due to the nature of their work, caring for patients with life-threatening illnesses. The emergence of professional burnout among these nurses is influenced by several factors, highlighting the importance of identifying protective and risk factors to mitigate its impact. This study aims to identify burnout profiles and protective socio-demographic and work-related patterns associated with reduced burnout among oncology nurses.

**Methods:**

A cross-sectional study was conducted with 150 oncology nurses at a specialized hospital exclusively dedicated to adult oncology treatment in Portugal. Data collection included a self-administered questionnaire incorporating the validated Portuguese version of Maslach Burnout Inventory (MBI). Statistical analyses were performed using SPSS and machine learning tools, specifically KMeans clustering and Random Forest algorithms.

**Results:**

Six protective patterns against burnout were identified, characterized by conditions of permanent contracts, work-life balance, and supportive work environments. Moreover, factors such as holding management roles and being a parent of two or more children might even be protective in some circumstances, suggesting a nuanced relation between personal and professional factors. Machine learning analyses made apparent the unpredictability of burnout and highlighted the critical role of protective factors in mitigating its impact.

**Conclusions:**

This study underscores the importance of resilience-building strategies and promoting protective factors, such as job stability, learned experience, and adequate rest, to reduce burnout risk among oncology nurses. Future research should validate these findings through hypothesis-driven analyses to inform targeted and context-specific burnout prevention programs.

**Clinical trial number:**

Not applicable.

**Supplementary Information:**

The online version contains supplementary material available at 10.1186/s12912-025-03277-5.

## Background

Burnout syndrome is a significant occupational issue for oncology nurses, impacting both their physical and mental health, as well as the quality of care they provide [1]. It is particularly prevalent among healthcare professionals working in emotionally demanding fields, such as oncology [2–4], and is now recognized in the International Classification of Diseases (ICD-11) under the code QD85 [5]. The nature of oncology nursing, which often involves providing care to individuals with advanced or life-threatening illnesses, contributes to sustained emotional strain and psychological fatigue [6, 7]. These demands place oncology nurses at increased risk of developing burnout, underscoring the urgent need for better understanding of how the syndrome manifests in this workforce [8].

Burnout is defined as a psychological syndrome resulting from chronic job-related stress, characterized by emotional exhaustion (EE), depersonalization (DP) (cynicism), and reduced personal accomplishment (PA). This three-dimensional model, as conceptualized by Maslach, reflects the dynamic interaction between individual stress responses and the broader social context [9]. The Maslach Burnout Inventory (MBI), developed to measure this construct, remains the most widely used instrument in healthcare settings [10].

While the prevalence of burnout among oncology nurses has been well-documented, estimates vary substantially depending on the setting, population and instruments used. For a example a meta-analysis of nearly 10,000 oncology nurses reported rates of 30% for emotional exhaustion, 15% for depersonalization, and 35% for low personal accomplishment [3]. In a Portuguese study by Paiva et al. [11], 8.9% of nurses were classified as experiencing burnout using a two-dimensional criterion, while only 1.3% met the full three-dimensional criteria. These disparities reflect the complexity of burnout and its dependence on contextual and methodological factors.

Previous research has primarily focused on identifying risk factors, such as younger age, high workload, and poor communication skills [3, 12]. However, fewer studies have examined the how socio-demographic and work-related variables interact to create profiles of vulnerability and protection. Rather than isolating personal traits, recent perspectives emphasize the importance of structural and contextual dimensions in shaping work environments that are more sustainable and conducive to well-being [13]. Understanding these patterns is essential for developing systems-level strategies to support oncology nurses and reduce burnout risk.

Recent approaches to burnout research also highlight the value of identifying distinct profiles or patterns of vulnerability and protection, rather than relying solely on variable-by-variable associations [14]. Such pattern-based perspective recognizes the complexity of how personal, professional, and organizational factors may interact [15]. In this context, machine learning techniques offer a valuable means of identifying data-driven burnout profiles that may remain undetected through traditional statistical approaches [16]. These methods can help reveal latent configurations of protective characteristics that support the well-being of oncology nurses.

### Portuguese adaptation of the Maslach burnout inventory to the Portuguese nursing context

The Maslach Burnout Inventory – Human Services Survey (MBI-HSS) is widely used to measure burnout among healthcare professionals. It assesses burnout across three dimensions and is tailored to human service fields, including healthcare, social work, and education [17].

In Portugal, several studies have assessed the MBI-HSS in contexts. Marôco et al. [18] confirmed its reliability and validity in a national sample, with strong reliability for Emotional Exhaustion (α = 0.87), moderate for Depersonalization (α = 0.72), and good for Personal Accomplishment (α = 0.82). A five-item short version for each sub-scale showed excellent model fit (CFI = 0.957, RMSEA = 0.027).

In the context of nursing, Laranjeira [19] validated 20 of the original 22 items for Portuguese nurses, reflecting some variability. da Fonte [20] reported high consistency for Emotional Exhaustion (α = 0.905) though two items from the other subscales were excluded to improve reliability (α = 0.799). Santos [21] initially identified seven factors explained 65.9% of the variance, but a forced three-factor solution reaffirmed the original MBI structure, with Emotional Exhaustion (α = 0.797) and Depersonalization (α = 0.763) showing satisfactory reliability.

Focusing on oncology nurses, Sá [22] found that while the MBI’s core dimensions remained relevant, some adjustments were needed. Item 15 from the Depersonalization subscale was removed due to poor performance, reducing the scale to 21 items. Emotional Exhaustion remained the most reliable dimension (α = 0.85), while Depersonalization showed lower reliability (α = 0.52). These findings underscore the need for context-sensitive applications of the MBI in oncology settings in Portugal.

### Research problem

Burnout is widely recognized as a serious concern among oncology nurses, yet how socio-demographic and work-related characteristics contribute to vulnerability or resilience remains insufficiently understood. Variations across care settings, workloads, and personal circumstances, highlight the need for deeper insight into the patterns that may protect against burnout in this workforce. Accordingly, this study aims to identify burnout profiles among oncology nurses and explore the socio-demographic and work-related characteristics associated with protective patterns that may mitigate burnout. The study’s research question is what protective patterns, based on socio-demographic and work-related characteristics, are associated with reduced burnout among oncology nurses?

## Methods

The present study resports a secondary analysis of data originally collected within a broader research project on the prevalence of burnout among health professionals, including oncology nurses, working in a hospital dedicated to cancer care in the Centre Region of Portugal. The original project followed a quantitative, exploratory, cross-sectional design.

The Strengthening the Reporting of Observational Studies in Epidemiology (STROBE-Statement) (Supplementary file A) was followed to report this manuscript.

### Inclusion and exclusion criteria

Data for this secondary analysis were drawn from a broader study conducted in a Portuguese tertiary hospital dedicated exclusively to adult oncology care. The original study targeted all healthcare professionals in the institution, with inclusion criteria being: aged ≥ 18 years, employed at the hospital, and capable of understanding the study purpose and providing informed consent. Professionals with psychiatric disorders or unwilling to participate were excluded.

The recruitment was conducted during regular staff meetings. The staff meetings were systematically planned department-level gatherings that included all healthcare professionals working in direct patient care at that department. During these meetings, the study was introduced by the research coordinator, who also distributed the data collection materials. Eligibility criteria were verified through hospital staffing records prior to the meetings. Healthcare professionals with a self-reported psychiatric diagnosis or who declined participation were excluded, consistent with the broader project’s inclusion criteria. For the current analysis, only nurses working in direct patient care were considered. Of the 216 eligible nurses, 150 completed the survey, yielding a response rate of 69.4%.

### Data collection

The study adhered to the principles of the Helsinki Declaration and was approved by the ethics committee of the institution where the research was conducted (Register number TI02/2017). Additionally, appropriate authorization was obtained from the original authors to use the MBI for research purposes. The data collection materials were individually distributed by the project’s coordinator during the staff meetings in sealed envelopes, each accompanied by a letter explaining the nature and objectives of the study and ensuring data confidentiality. Participants were given the flexibility to complete the questionnaires during work hours or at home and were instructed to return them in sealed envelopes. This process ensured minimal disruption to their professional duties while maximizing participation and maintaining confidentiality.

After obtaining informed consent, data were collected using a protocol specifically designed for this study. The protocol included a sociodemographic questionnaire covering key participant information such as age, gender, marital status, number of children, education level, professional category, years of work, weekly workload, night shifts, employment contract type, management position, and hours of sleep per day, and the MBI-HSS.

The MBI-HSS consists of 22 items measuring burnout across three dimensions: EE, DP, PA. Responses are given on a 7-point Likert scale ranging from 0 (Never) to 6 (Every day), reflecting the frequency with which the respondent experiences these feelings.

The interpretation of burnout is based on cut-off scores derived from normative data. High levels of burnout are indicated by high scores in EE and DP, and low scores in PA. Conversely, low burnout is characterized by low scores in EE and DP, and high scores in PA.

The cut-offs for each dimension typically divide scores into low, moderate, and high categories. For EE, scores between 0 and 16 are considered low, 17 to 26 are moderate, and scores above 27 are high. In the DP dimension, scores from 0 to 6 are classified as low, PA Personal Accomplishment, low scores are 39 and above, moderate scores range from 32 to 38, and high burnout is indicated by scores between 0 and 31.

Burnout is considered severe when individuals score high in EE and DP, and low in PA. The MBI does not generate a single burnout score but provides a profile across these three dimensions, giving a comprehensive view of the individual’s burnout experience [17].

### Data analysis

Data were analyzed using SPSS version 24.0. Descriptive statistics were calculated for socio-demographic and work-related variables, and for the items of the Maslach Burnout Inventory (MBI). Missing data analysis was performed for all items.

The software also assisted psychometric assessment of the MBI-HSS. To complement this analysis, machine learning, specifically using Random Forest models, was applied to further examine the MBI structure and assess the relationships between individual items and burnout dimensions, exploring their alignment with theoretical assumptions.

Additionally, machine learning was used to identify the most important socio-demographic and work-related variables contributing to burnout. Moreover, KMeans clustering was employed to group nurses based on their burnout profiles derived from the MBI dimensions, enabling the identification of distinct burnout patterns. No a priori sample size calculation was performed for the purpose of the machine learning analyses.

### Assessment of MBI structure in the study sample

Given ongoing evidence of variation in factor structure and item retention depending on the target population, the following procedures were conducted to assess the psychometric properties to confirm the the MBI’s dimensional structure and reliability in this sample of oncology nurses.

Reliability analysis was conducted to assess internal consistency, using Cronbach’s alpha for the total MBI scale and its dimensions: EE, DP, and PA. Alpha values between 0.70 and 0.79 were considered indicative of acceptable reliability, 0.80 to 0.89 as good, and values ≥ 0.90 as excellent.

Construct validity was evaluated through two methods: (i) item-construct and item-total correlations, and (ii) exploratory factor analysis (EFA). Correlations were categorized as inadequate if they were below 0.20, adequate between 0.20 and 0.34, good within the range of 0.35–0.49, and excellent at 0.50 or above [23].

The suitability of the data for factor analysis was confirmed using the Kaiser-Meyer-Olkin (KMO) measure of sampling adequacy and the Bartlett’s test of sphericity. The EFA was conducted using Principal Component Analysis with Varimax rotation to extract underlying factors. Factors were retained based on Eigenvalues ≥ 1 and the Scree Plot [24].

### Machine learning analyses

To address the research question, we applied two machine learning techniques: KMeans clustering and Random Forest classification. KMeans, an unsupervised learning algorithm, grouped oncology nurses based on their scores in the MBI dimensions, generating distinct burnout profiles without relying on predefined labels.

Following this, the Random Forest algorithm, a supervised learning technique, was used to classify nurses into burnout and non-burnout clusters. Random Forest builds an ensemble of decision trees, allowing for accurate classification by aggregating results across multiple trees. A key feature of a Random Forest is explainability, providing insights into which MBI dimensions contribute most to classifying burnout, by visualizing each individual decision tree.

These methods were chosen due to their increasing application in healthcare research, particularly in exploring complex, non-linear relationships in psychological outcomes and occupational health [25, 26]. Their capacity to reveal latent structures and handle high-dimensional data makes them particularly suitable for exploratory analyses in fields such as burnout and mental health (e.g [27, 28]). In the present study, they offered a data-driven way to identify meaningful profiles that might be missed by traditional statistical approaches.

The decision trees were interpreted based on two main criteria: (1) Cluster Homogeneity – clusters with lower Gini values were considered more homogeneous and distinct, making them more reliable for analysis; and (2) Sample Size – clusters grouping five or more nurses were prioritized to ensure sufficient sample size, reducing the impact of random variation and providing a more stable basis for identifying meaningful patterns within the data.

In a final step, a Random Forest was constructed to analyze, additionally, socio-demographic and work-related variables, to identify potential protective and risk factors associated with burnout. This step provided an understanding of how these variables influence burnout risk. All analyses were conducted on a split sample, with 70% of the data used for training and 30% for testing, ensuring the machine learning algorithm remained unbiased, free from overfitting, and blind to theoretical cut-off values.

Model performance was evaluated using accuracy, sensitivity, and specificity metrics. Accuracy assessed the overall correct classification rate, while sensitivity measured the model’s ability to correctly identify cases of burnout. Specificity evaluated the model’s effectiveness in correctly identifying non-burnout cases.

To assess clusters’ validity, the data from the significant decision trees, cf., the Gini and Sample size criteria, were compared with existing literature and analyzed in Excel against the burnout prevalence within the study sample.

## Results

### Sample characteristics

The study involved 150 oncology nurses, representing 69,4% of the hospital’s nursing staff (Table 1). The sample comprised 123 women, with a mean age of 39.82 years (SD = 8.86), and 59.3% were married. Nurses reported working a mean of 36.47 h per week (SD = 4.18) in oncology services, and the majority (70%) had been employed at the institution for over 10 years. Most participants had a permanent employment contract (96.7%) and worked night shifts (59.3%). Additionally, 44% reported having secondary work activities. Of the total sample, 66% had at least one child, and 76.7% reported sleeping the recommended number of hours per night.

**Table 1:** Descriptive statistics of oncology nurses

Variable		M	Md	SD	Min	Max
Age (years)		39.82	39	8.86	23	61
Workload (hours *per* week)		36.47	40	4.18	30	42
Number of Children (Son/Daughther)		1.05	1	0.9	0	4
**Variable**		**n (***N* = **150)**		**%**		
Gender	Female	123		82		
	Male	27		18		
Marital Status	Single	46		30.70		
	Married	89		59.30		
	Divorced	14		9.3		
	Widow	1		0.7		
Be a parent	Yes	99		66		
	No	51		34		
Night Shift	Yes	89		59.3		
	No	60		40		
Permanent Contract	Yes	145		96.7		
	No	5		3.3		
Management Functions	Yes	21		14		
	No	129		86		
Extra work activities	Yes	66		44		
	No	84		56		
Time in Institution (years)	≤ 5	25		16.7		
	6–10	20		13.3		
	> 10	105		70		
Sleeping hours (per day)	≥ 6	31		20.7		
	6–8	115		76.7		
	> 8	4		2.7		

M = Mean; Md = Median; SD = Standard Deviation; Min = Minimum; Max = Maximum; n = Sample size; % = Percentage

### Psychometric results

Reliability analysis revealed that the MBI-HSS is a homogeneous and valid instrument for measuring burnout in this sample (Table 2). The Emotional Exhaustion subscale showed strong consistency (α = 0.90), with item-total correlations ranging from 0.579 to 0.811. The highest correlation was for Item 8 (“I feel exhausted by my work”), and its removal would reduce the reliability (α = 0.880). For the Depersonalization subscale, reliability was acceptable (α = 0.78), with correlations between 0.468 and 0.612. Item 5 had the highest correlation, while Item 22 showed the lowest. Yet, removing Item 22 would reduce the alpha to 0.761. The Personal Accomplishment subscale had good consistency (α = 0.85), with correlations ranging from 0.291 to 0.762. Item 18 contributed the most to the scale’s reliability, while removing Item 4 would slightly increase the alpha to 0.868.

**Table 2:** Item homogeneity statistics and internal consistency coefficients

Dimensions and Items	Mean (SD)^1^	Item-construct correlation	*r*	Cronbach’s α if item deleted
**Emotional Exhaustion (α = 0**,**90)**				
1. I feel emotionally drained from my work.	1,63 (1,53)	,739*	,502	,892
2. I feel used up at the end of the workday.	3,69 (1,51)	,751*	,580	,893
3. I feel fatigued when I get up in the morning and have to face another day on the job.	2,90 (1,55)	,750*	,613	,891
6. Working with people all day is really a strain for me.	1,99 (1,73)	,676*	,482	,898
8. I feel burned out from my work.	2,91 (1,66)	,861*	,691	,880
13. I feel frustrated by my job.	2,25 (1,66)	,837*	,657	,882
14. I feel I’m working too hard on my job.	3,47 (1,72)	,729*	,502	,895
16. Working with people directly puts too much stress on me.	2,23 (1,69)	,701*	,453	,896
20. I feel like I’m at the end of my rope.	1,91 (1,71)	,737*	,453	,893
**Depersonalization (α = 0**,**78)**				
5. I feel I treat some recipients as if they were impersonal objects.	,75 (1,22)	,635*	,413	,726
10. I’ve become more callous toward people since I took this job.	1,49 (1,76)	,760*	,397	,729
11. I worry that this job is hardening me emotionally.	1,89 (1,76)	,758*	,326	,740
15. I don’t really care what happens to some recipients.	,91 (1,29)	,676*	,428	,720
22. I feel recipients blame me for some of their problems.	1,31 (1,48)	,614*	,276	,761
**Personal Accomplishment (α = 0**,**85)**				
4. I can easily understand how my recipients feel about things.	4,66 (1,29)	,345*	,228	,868
7. I deal very effectively with the problems of my recipients.	4,19 (1,19)	,650*	,380	,836
9. I feel I’m positively influencing other people’s lives through my work.	4,63 (1,39)	,670*	,382	,835
12. I feel very energetic.	3,51 (1,48)	,633*	,389	,850
17. I can easily create a relaxed atmosphere with my recipients.	4,51 (1,33)	,783*	,579	,819
18. I feel exhilarated after working closely with my recipients.	4,15 (1,32)	,818*	,635	,815
19. I have accomplished many worthwhile things in this job.	3,99 (1,47)	,754*	,481	,827
21. In my work, I deal with emotional problems very calmly.	4,04 (1,45)	,762*	,475	,826

^1^ SD: Standard Deviation; * The correlation is significant at the 0.01 level (two-tailed)

The sample size adequacy and the sufficiency of the correlations between items to perform factor analysis were determined by the KMO value of 0.887, and a significant Bartlett’s test of sphericity (χ² = 1,715.309, *p* < 0.001) [24].

Based on eigenvalues greater than 1, a four-factor solution was retained, explaining a total of 62.17% of the variance. Factor 1, primarily representing Emotional Exhaustion, included items with strong loadings, such as Item 8 (“I feel exhausted by my work,” loading = 0.859) and Item 2 (“At the end of the workday, I feel exhausted,” loading = 0.802). Factor 2, associated with Personal Accomplishment, featured items such as Item 18 (“I feel invigorated after working closely with my patients,” loading = 0.805) and Item 17 (“I can easily create a relaxed atmosphere with my patients,” loading = 0.761). Factor 3 corresponded to Depersonalization, with Item 5 (“I treat some patients as impersonal objects,” loading = 0.735) and Item 11 (“I am worried this job is hardening me emotionally,” loading = 0.698) loading strongly.

Item 4 (“I can easily understand how my patients feel”) loaded on Factor 4 with a high value of 0.809, but it was the only item loading on this factor. Despite this, the high communality of 0.727 and lack of cross-loadings above 0.3 justified its retention, given its theoretical importance.

The Random Forest model exploring the relationships between individual items and burnout dimensions indicated that the Random Forest analysis effectively replicated the theoretical structure of the MBI.

The results confirmed the robustness of the MBI’s theoretical dimensions and their reliability in assessing burnout in oncology nurses, supporting the use of the original structure and corresponding cut-off values in this study. Although the instrument has previously been validated in the Portuguese healthcare context, no item modification or cultural adaptation was applied. The full 22-item version of the MBI was used in its original form.

### Burnout profiles and protective and risk factors

**Fig. 1:**
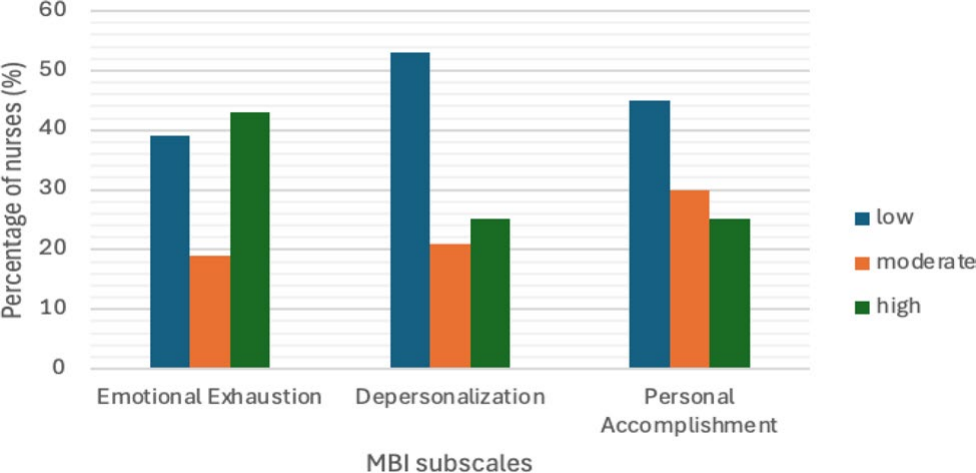
EE, DP and PA in the study sample

From the descriptive statistics performed to analyze burnout among oncology nurses, the MBI subscales revealed significant insights into their emotional well-being (Fig. 1). The mean EE score was 22.99, with 43% of nurses scoring in the high burnout range (EE ≥ 27), 19% in the moderate range, and 39% reporting low emotional exhaustion. DP, which had a mean score of 6.36, was high in 25% of nurses, moderate in 21%, and low in 53%, indicating that most nurses maintained a connection with their patients. PA, with a mean score of 33.68, showed that 25% of nurses had low PA (PA < 34), 30% had moderate PA, and 45% had high PA, suggesting that a significant proportion of nurses still felt competent and effective in their roles.

Based on the established burnout criteria, i.e., high emotional exhaustion and/or high depersonalization, combined with low personal accomplishment, the overall prevalence of burnout in this sample of oncology nurses was 17.3%.

**Fig. 2:**
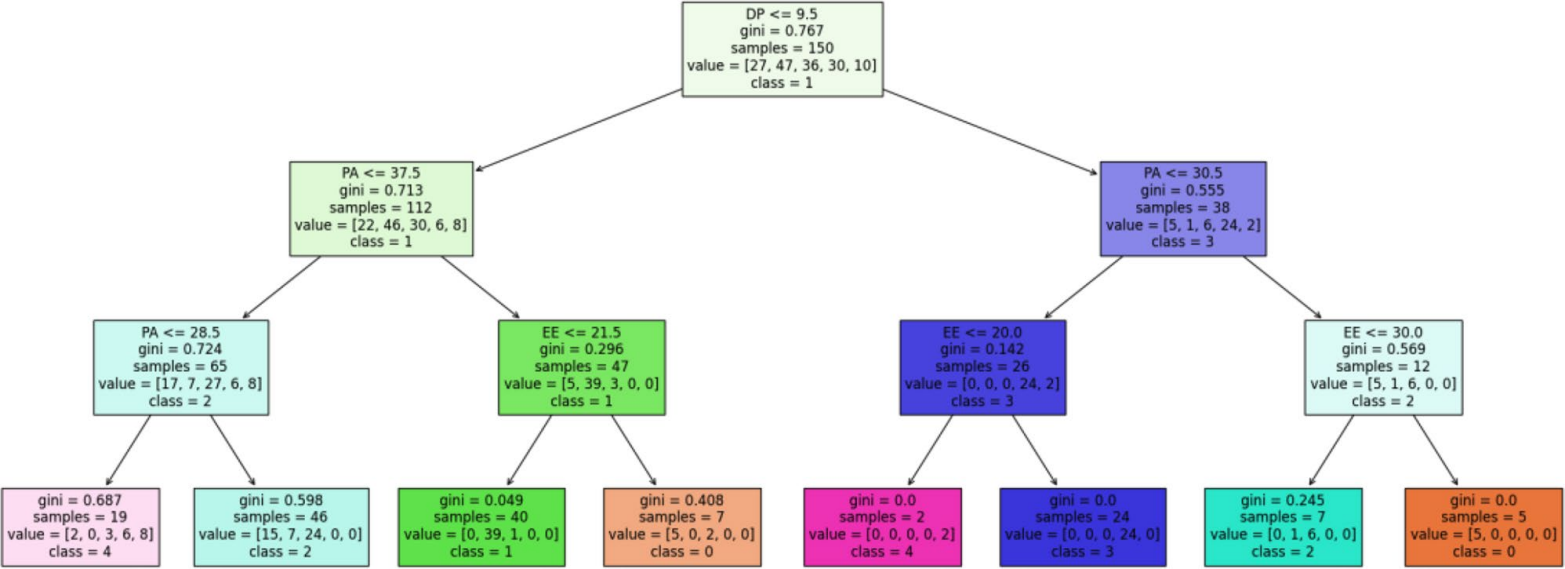
KMeans clusters interpreted using a Decision Tree model

The KMeans algorithm grouped the sample of oncology nurses based on their levels of PA, DP, and EE. Five clusters were specified for the KMeans algorithm. Cluster 3 contained 24 nurses whose scores were consistent with the burnout thresholds defined by the theoretical model (i.e., DP > 9.5, PA ≤ 30.5, EE > 20), aligning closely with the established burnout cut-offs (Fig. 2). The remaining four clusters correspond to burnout-free nurses.

**Fig. 3:**
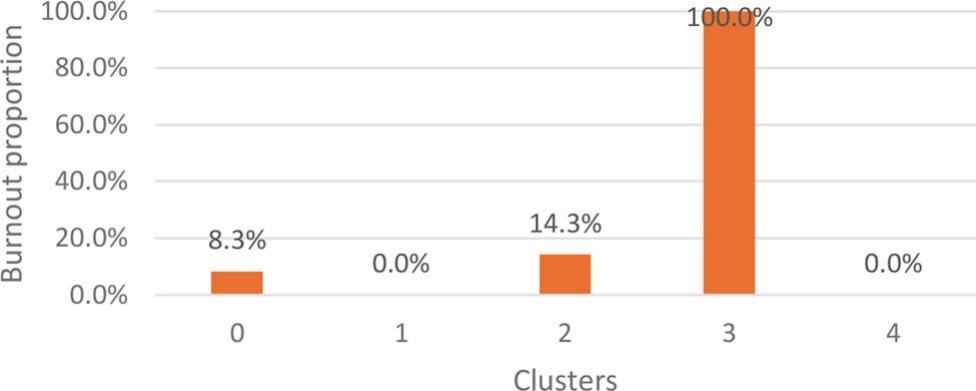
Burnout proportion in the KMeans clustering

Additionally, two other cases were split, with 8.3% (*n* = 12) assigned to Cluster 0 and 14.3% (*n* = 7) to Cluster 2 (Fig. 3). These groupings were based on their burnout profiles, as calculated by KMeans.

Following this, the Random Forest algorithm was used to classify the identified clusters based on socio-demographic and work-related variables, specifically to classify nurses as belonging to Cluster 3 (burnout) or to any of the burnout-free clusters. Model performance showed an accuracy of 78%, with a sensitivity of 0% and a specificity of 100%. This implies that the model was highly effective in identifying non-cases of burnout, correctly classifying all nurses without burnout. These performance metrics indicate that the machine learning model revealed valuable insights into protective factors associated with non-burnout profiles among oncology nurses, while it did not recognize any risk factors contributing to burnout.

Random Forest models consisting of three decision trees were trained. Despite exploring models with higher tree numbers and depths, this ensemble provided the best balance between stability and simplicity, because greater numbers of trees and depths did not improve the accuracy metrics.

**Fig. 4:**
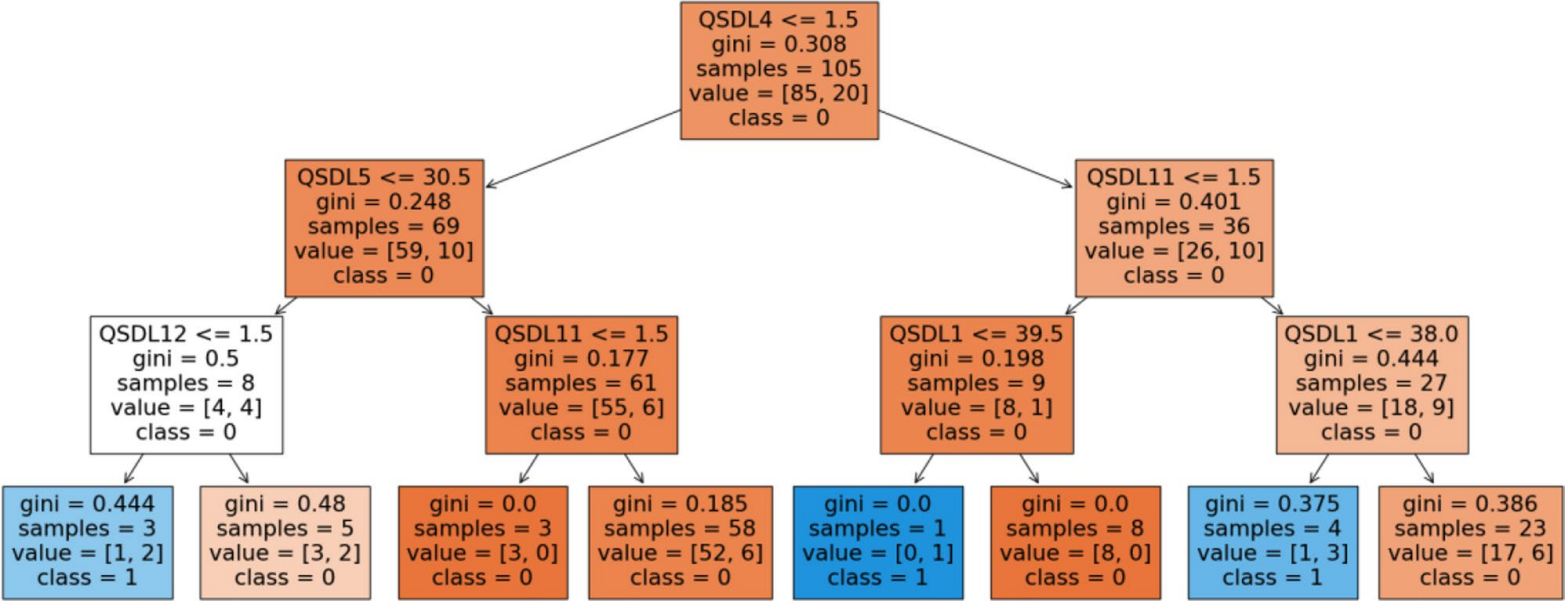
Decision-tree one, distinguishing the number of children (QSDL4), the workload (QSDL5), Management duties (QSDL 11), and Age (QSDL1)

Decision tree number one (Fig. 4) reflects two classes: cases of burnout in blue and non-burnout cases in orange. Given the absence of model’s sensitivity, only the orange class was analyzed. The root node split is based on the *number of children* (QSDL4), with a threshold of 1.5, indicating that this variable plays a critical role in the initial classification. Samples were divided into two groups: those with QSDL4 ≤ 1.5 and those with QSDL4 > 1.5. At the second level, further splits are observed. For instances where QSDL4 ≤ 1.5, the next split occurs on *workload* (QSDL5) with a threshold of 30.5, while for QSDL4 > 1.5, the split is determined by *management duties* (QSDL11), also at 1.5. These subsequent divisions highlight the importance of QSDL5 and QSDL11 in refining the classification further. The terminal nodes of the first decision tree have Gini values close to 0, indicating high purity and well-separated classifications. Specifically, in the case where QSDL4 > 1.5 and QSDL511 ≤ 1.5 and *Age* (QSDL1) > 39.5, the Gini index is 0, indicating no misclassification at that node.

**Fig. 5:**
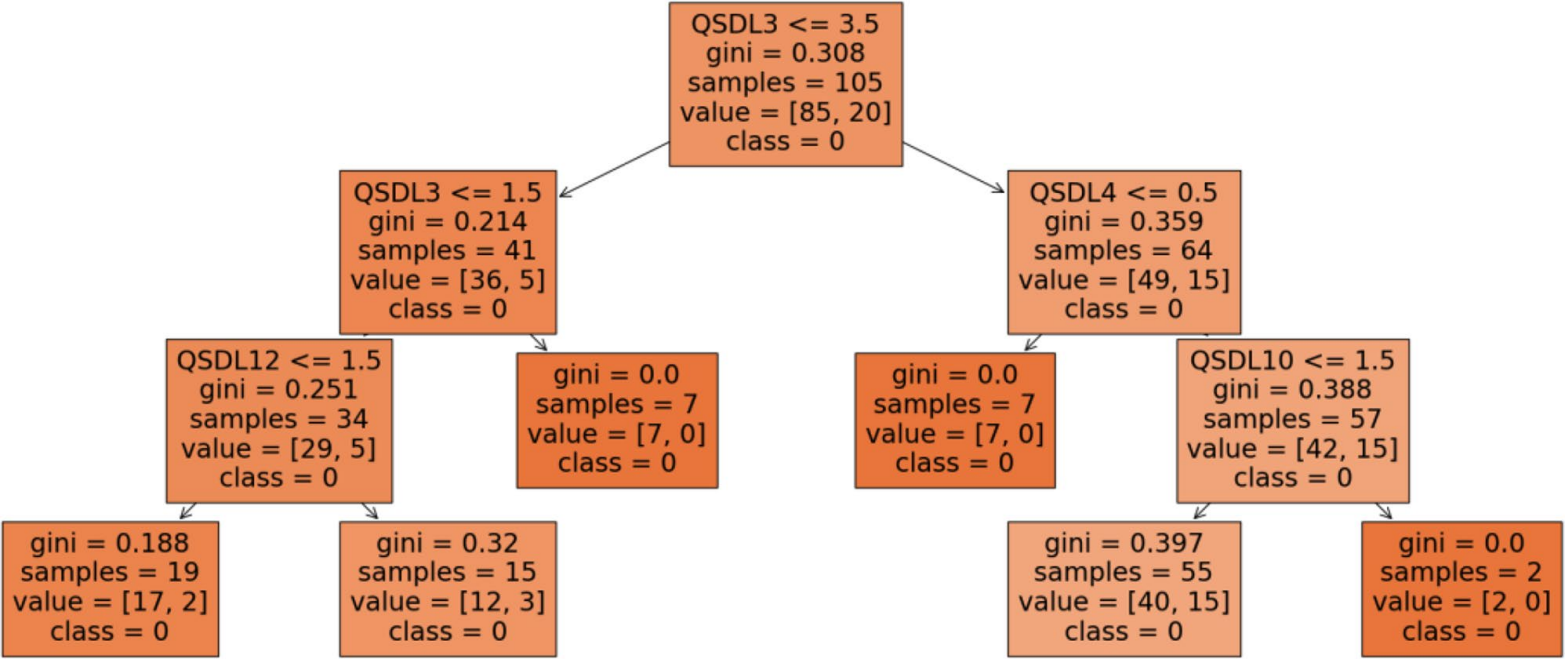
Decision-tree one, distinguishing the civil status (QSDL3), number of children (QSDL4), Leisure activities (QSDL12) and the type of job contract (QSDL10)

In the second decision tree (Fig. 5), the root node is determined by the *civil status* (QSDL3), with a split at 3.5, suggesting that this variable is central in distinguishing between non-burnout profiles at the top level. For the branch where QSDL3 ≤ 3.5, the next split occurs based on *participation in leisure activities* (QSDL12) at 1.5, indicating its relevance in further distinguishing samples. On the other side, when QSDL3 > 3.5, the model uses QSDL4 at 0.5 to further split the data. Altogether three terminal nodes exhibit low Gini values. Where QSDL3 ≤ 1.5 and QSDL12 ≤ 1.5, the Gini index reaches 0.19. If QSDL3 > 1.5, Gini equals 0. Similarly, when focusing on the right branch split, where of QSDL3 > 3.5 and QSDL4 ≤ 0.5, Gini equals 0. On the other side, if QSDL4 > 0.5, then the *type of job contract* (QSDL10) at 1.5 further distinguishes the classes. Although reaching a Gini of 0, the sample only comprises two nurses.

**Fig. 6:**
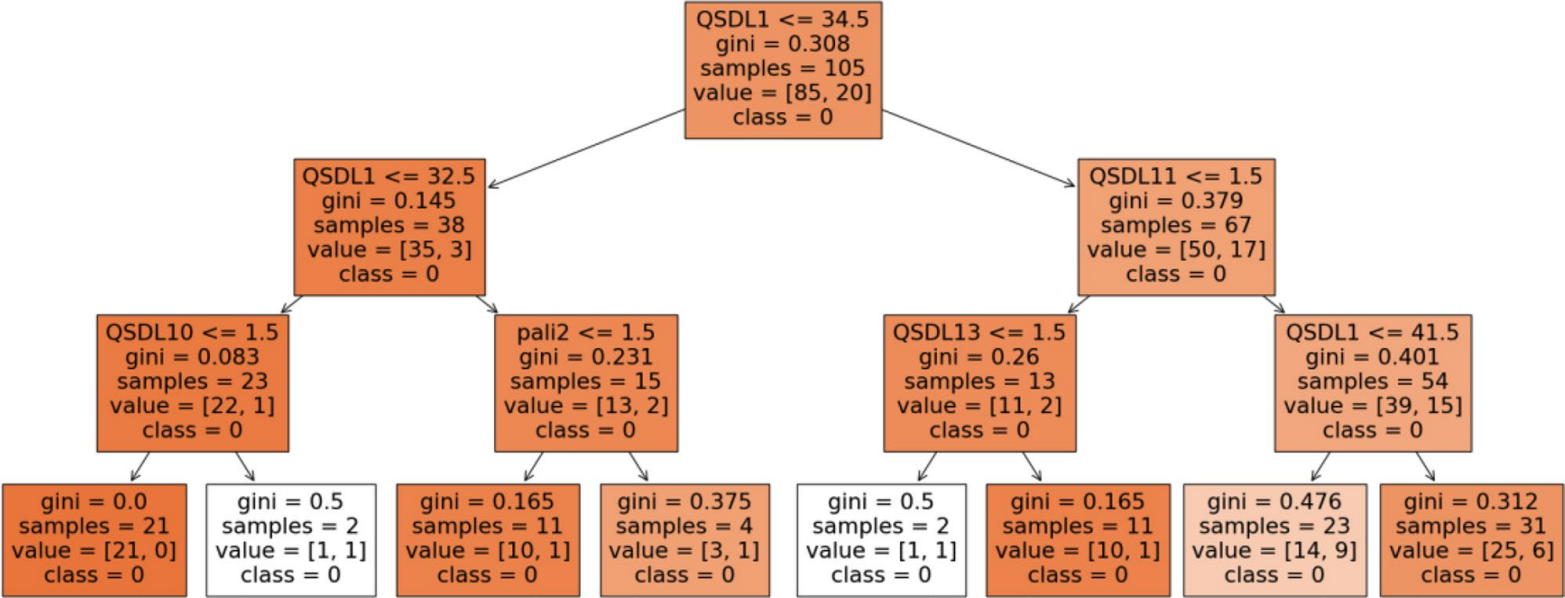
Decision-tree one, distinguishing Age (QSDL1), the type of job contract (QSDL10), the working setting (pali2), Management duties (QSDL 11), and Sleeping hours (QSDL13)

The third decision tree (Fig. 6) begins with a root node split on QSDL1, with a threshold of 34.5, indicating that *age* is the primary driver of classification in this model. Cases where QSDL1 ≤ 34.5 are split further into two branches. On the left branch, the next critical split is on QSDL10 at 1.5. The terminal right node in this branch demonstrates high classification accuracy, with Gini equalling 0 and a sample of 21 nurses. If QSDL1 greater than 32.5 but lower than 34.5, then the *working setting* (pali2) follows to further split the sample at 1.5, leading to a terminal node of Gini at 0.17 and a sample of 11 nurses. On the right side, for instances where QSDL1 > 34.5, the tree splits again on QSDL11 at 1.5, and further divisions are based on QSDL13 and QSDL1 with thresholds of 1.5 and 41.5, respectively. As with the left branch, the terminal nodes here also exhibit low Gini values, indicating highly effective classifications. For example, where QSDL1 > 34.5, QSDL11 ≤ 1.5 and Sleeping hours (QSDL13) > 6 h (threshold > 1.5), Gini lowers to 0.17 with a sample of 11 nurses.

Proceeding to the validity assessment in Excel, the prevalence of burnout was determined in the clustered samples. Clusters were determined valid if their burnout prevalence was inferior to the overall sample (i.e., ≤ 17,3%), given the performance model results showing the absence of predictive burnout capability (cf., sensitivity = 0%) but effective prediction of non-cases of burnout (specificity = 100%).

After determining the prevalence of burnout in the clustered samples, only six patterns were considered valid (Table 3). Two patterns failed validation as their burnout prevalence was greater than the total sample burnout prevalence (17.3%), i.e., 20% and 23%, respectively.

**Table 3:** Valid clusters of protective patterns based on socio-demographic and work-related variables

Pattern	Model Gini & sample	Cluster size	Burnout Prevalence
*Tree 1*,* Pattern A*: Number of children ≤ 1.5; workload > 30,5 h; No management duties	0.19; 58	75	12%
*Tree 1*,* Pattern B*: Number of children > 1.5; Management duties; Age > 39.5	0.00; 8	10	10%
*Tree 2*,* Pattern C*: Single; Leisure activities	0.19; 19	26	11,5%
*Tree 2*,* Pattern D*: Married; No children	0.00; 7	9	0%
*Tree 3*,* Pattern E*: Age ≤ 32.5; Permanent position	0.00; 21	29	3,4%
*Tree 3*,* Pattern F*: Age > 34.5; Management duties; sleeping hours > 6 h	0.17; 11	15	13,3%

The analysis of the decision trees identified several significant patterns highlighting protective factors of burnout in the current sample of oncology nurses. Pattern A includes half of the total sample (75 nurses). This pattern, characterized by nurses with only one or no children, working more than 30.5 h per week, and holding no management duties, showed a burnout prevalence of 12%. Compared to the overall burnout prevalence of 17.3% in the sample, Pattern A prevalence reveals a reduction of 5.3% points, equivalent to a 30.6% decrease in burnout prevalence in the overall sample. This indicates that specific work and personal characteristics—such as the absence of management duties and certain family structures—may serve as protective factors against burnout within this group.

Furthermore, Pattern D revealed a burnout-free group consisting of 9 nurses who are married and have no children. The 0% burnout prevalence in this cluster suggests protective factors associated with marital status (i.e., being married) combined with the absence of children. It must be highlighted that lacking these protective factors does not necessarily lead to higher burnout risk (for instance, in Pattern B having two or more children is protective). Therefore, Pattern D can be especially helpful in prioritizing other groups for preventive strategies.

Additionally, Pattern E identified younger nurses aged ≤ 32.5 years in permanent positions as another group with low burnout risk. With 29 nurses in this cluster and a burnout prevalence of just 3.4%. Compared to the overall burnout prevalence of 17.3% in the sample, Pattern E demonstrates a difference of 13.9% points, which would be equivalent to an 80.3% reduction in burnout prevalence in the overall sample. This pattern highlights that job stability in younger nurses may serve as a protective factor against burnout.

Pattern B identifies a group of nurses who are in management positions, have two children or more, and are aged over 39.5 years. This cluster, consisting of 10 nurses, has a relatively low burnout prevalence of 10%. Compared to the overall burnout prevalence of 17.3% in the sample, Pattern B shows a decrease of 7.3% points, which would be equivalent to 42.2% reduction in burnout prevalence in the overall sample. This suggests that the age and experience of these nurses may act as protective factors, helping them to manage burnout more effectively.

Pattern C groups 26 nurses who are single and engage in leisure activities, with a burnout prevalence of 11.5%. Compared to the overall burnout prevalence of 17.3% in the sample, Pattern C exhibits a difference of 5.8% points, which would be equivalent to a 33.5% reduction in burnout prevalence in the overall sample. Similarly to Pattern D, this pattern can help prioritizing other groups for burnout prevention while also suggesting a moderate role of leisure activities as a protective factor.

Lastly, Pattern F highlights a cluster of 15 nurses who are aged over 34.5 years, have management duties, and sleep for more than 6 h per night. The burnout prevalence in this group is 13.3%. Compared to the overall burnout prevalence of 17.3% in the sample, Pattern F shows a difference of 4% points, which would correspond to a 23.1% reduction in burnout prevalence in the overall sample. Similarly to Pattern B, these results indicate that the combination of age and experience may help nurses to handle burnout, while also highlighting the importance of sleep.

## Discussion

Oncology nurses face the constant challenge of providing emotional support to patients and are frequently exposed to their suffering, leading to chronic stress that may contribute to burnout syndrome [29]. In this study, the machine learning analysis revealed distinct patterns of potential protective factors that appear that appear to shape burnout profiles among oncology nurses. While no clear risk factors emerged, several configurations were identified as significant in reducing burnout risk, offering valuable insights for targeted preventive strategies.

The burnout prevalence identified in our study aligns with other research conducted among Portuguese oncology nurses, but some variations are evident when comparing findings from studies such Sá [22] and subsequent theses, which reported similar burnout levels but highlighted differences in specific burnout dimensions. For example, Sá [22] found that emotional exhaustion was the most prevalent burnout symptom in oncology nurses, with the depersonalization dimension being less pronounced, possibly reflecting the emotional nature of oncology care. In contrast, other studies conducted among Portuguese nurses, such as those by da Fonte [20] and Santos [21], observed moderate levels of burnout but with significant variability in depersonalization and personal accomplishment scores.

These discrepancies in burnout rates may be attributable to differences in organizational settings and work conditions. It is known that environment where job demands are increasingly disproportionate to job resources, lead to high levels of burnout [30].

Cumbie et al. [31], for instance, noted slightly lower burnout levels in settings where emotional support systems were more robust. Additionally, factors such as workload, nurse-patient ratios, and the availability of coping resources could explain why some studies, including ours, report higher levels of emotional exhaustion [32]. The findings across these studies highlight the need for context-specific interventions tailored to the challenges faced by oncology nurses in Portuguese healthcare settings.

Altogether, the findings drawn from Patterns B and F reinforce that factors like age, experience, and adequate sleep may mitigate burnout. Evidence from a meta-analysis highlighted some factors that predispose to burnout, and are complementary to the findings described, for instance factors related with characteristics of the profession, as rotative shifts, workload, and to sociodemographic variables such as marital status, work experience, age and gender [33]. Although in this study the gender was not a significant variable, other studies also add gender as a predisposing burnout factor, namely belonging to female gender [34].

It is important to emphasize Patterns A, D, and E, which showed some variables that protect from burnout syndrome. In Pattern A (characterized by having one or no children, working > 30.5 h per week, and having no management duties) particularly highlights the absence of managerial roles as a protective factor. Managers are tasked with ensuring high-quality care, which involves active problem-solving and requires future resilience and confidence from leaders under highly stressful conditions. These challenges often lead to burnout, affecting both job satisfaction and leadership effectiveness [35, 36].

Pattern D (characterized by being married and having no children) highlights the significance of personal support without the responsibilities of parenthood. The relation among these factors is complex, as evidenced by the fact that Pattern B nurses have two or more children as a protective factor. This complexity can be associated to working parents facing numerous demands, including workload, night shifts, physical tiredness, low income, social demands [37–39].

Work and family are typically viewed as distinct yet interdependent domains, with boundaries that allow for some degree of permeability [40]. Studies have recommended the provision of work-life balance programs to improve nurses’ psychological well-being [40, 41]. This underscores the critical role of work-family-life balance in nurse’s mental well-being. Several factors have been identified as influencing this balance, including working conditions, workload, leave policies, remuneration, career development opportunities, job satisfaction and security, organizational commitment, family and social relationships, self-care, and broader public health challenges [42].

Furthermore, evidence indicate that individuals with strong social support and adaptive coping strategies tend to report lower levels of burnout [43, 44]. This suggests that strategies aiming at strengthening support networks and promote self-care are essential to preserve the mental health of healthcare professionals.

Pattern E, (Age ≤ 32.5; Permanent position) highlights the importance of creating work stability with permanent positions, namely for the youngest nurses. Such job stability may provide nurses with the confidence to invest in their personal lives, underscoring the importance of promoting permanent positions early in their careers. An integrative review [45], highlights the value of supporting nurses during undergraduate training and early professional by fostering realistic expectations and effective coping strategies to address the complex organizational and functional demands of nursing [45]. Additionally, it is further recognized that younger individuals are often the most impacted by shifts in the job market, the instability brought on by career transitions, and uncertainties about future career growth, which may increase their vulnerability to developing burnout [45].

Burnout among oncology nurses is closely linked to the high emotional and physical demands of caring for cancer patients, often resulting in severe emotional exhaustion and depersonalization [4]. The variability in burnout profiles identified in our study underscores the importance of developing interventions that address the unique stressors faced by oncology nurses. These findings suggest that interventions should move beyond generalized group approaches and embrace more tailored, person-centered strategies.

Person-centered care (PCC) principles advocate for strategies that are context-specific and adaptable to the individual’s emotional needs, which is particularly crucial in oncology settings where emotional resilience varies widely among nurses [46]. Nurses in high emotional exhaustion groups may benefit more from emotional support systems, while those in low personal accomplishment clusters may require professional development and recognition programs. The effectiveness of such supportive strategies is highly context-dependent, aligning with person-centeredness principles towards individualized plans that reflect both the care environment and the cultural context of the healthcare system [46].

These results align with broader research, which suggests that burnout interventions in oncology care must consider not only the individual nurse experience but also the broader organizational and emotional demands [31, 32]. A scoping review exploring the association between PCC and healthcare providers’ job satisfaction and work-related health supports this view, demonstrating that PCC interventions improve job satisfaction and reduce work-related stress through tailored, context-sensitive approaches [47]. As such, implementing person-centered, contextually tailored strategies is crucial to improve the well-being of oncology nurses.

This study has some limitations that should be acknowledged. First, although the MBI is widely used and validated for measuring burnout, it was the sole instrument used in this report. While adequate for measuring the core dimensions of burnout, it does not capture broader elements of occupational well-being, such as work engagement, job satisfaction, or perceptions of organizational culture (e.g. [48, 49]). The inclusion of complementary instruments could have offered a more comprehensive view of protective and contextual factors relevant to oncology nursing.

Secondly, while machine learning methods are well-suited for identifying complex patterns of burnout in multidimensional data [50], their performance can be affected by dataset size and variable distribution. In this study, the model achieved 100% specificity but 0% sensitivity, indicating strong accuracy in identifying non-burnout cases but limited ability to detect actual burnout. Similar limitations have been noted in other psychological studies applying machine learning techniques, where class imbalance or subtle feature differences can hinder sensitivity (e.g. [47, 51]). Although no a priori sample size calculation was conducted, measures such as sample splitting (70/30 train-test), performance evaluation (accuracy, specificity), confusion matrix determination, and Gini index analysis were applied to support the robustness of the exploratory findings. The patterns uncovered in the present study are acknowledge as exploratory and hypothesis-generating in nature.

Together, these limitations underscore the need for future studies to use larger, purposefully designed datasets and integrate complementary methodologies to further clarify and validate the protective factors identified.

## Conclusion

The findings of this study highlight the complexity of burnout risk among oncology nurses, demonstrating that burnout is not easily predicted by socio-demographic and work-related variables alone. Machine learning analysis revealed that protective factors seem to play a larger role in shaping burnout than specific risk factors. This suggests a nuanced interplay of individual, social, and professional circumstances that can influence burnout in unexpected ways, underscoring the unpredictability of burnout onset in this population.

Rather than pinpointing clear-cut risk factors, the results emphasize the value of fostering protective factors identified in specific nurse profiles. The presence of these protective elements, such as job stability in younger nurses, learned experience that can be instrumentally shared, and the alleviating effect of adequate sleep and leisure activities, appears pivotal in reducing burnout levels. These insights encourage a shift toward preventive strategies that prioritize protective factors within the work environment and beyond.

Given the unpredictable nature of burnout, these findings advocate for a proactive approach that focuses on reinforcing protective factors within the organizational and work environment. Tailoring systemic interventions to enhance job stability, work-life balance, and supportive workplace conditions offers a promising direction for promoting well-being and reducing burnout in oncology nursing.

Future research should focus on validating the protective patterns identified in this study within broader oncology nursing datasets, using hypothesis-driven statistical analyses. By testing predefined hypotheses concerning each protective factor’s impact on burnout, such studies could confirm the significance and generalizability of these findings across diverse nursing populations and settings. This validation step would provide clarity and interpretability, facilitating the translation of the insights on job stability, family support, and work-life balance into actionable strategies that can be systematically integrated into burnout prevention programs for oncology nurses.

## Electronic supplementary material

Below is the link to the electronic supplementary material.

Supplementary Material 1

Supplementary Material 2

## Data Availability

The datasets used and/or analyzed during the current study are available from the corresponding author upon reasonable request.

## Abbreviations

ICD-11: International Classification of Diseases
EE: Emotional exhaustion
DP: Depersonalization
PA: Personal accomplishment
MBI: Maslach Burnout Inventory
MBI-HSS: Maslach Burnout Inventory – Human Services Survey
STROBE-Statement: Strengthening the Reporting of Observational Studies in Epidemiology
